# An exploratory study to evaluate the utility of an adapted Mother Generated Index (MGI) in assessment of postpartum quality of life in India

**DOI:** 10.1186/1477-7525-6-107

**Published:** 2008-12-02

**Authors:** Jitender Nagpal, Rinku Sen Gupta Dhar, Swati Sinha, Vijaylakshmi Bhargava, Aarti Sachdeva, Abhishek Bhartia

**Affiliations:** 1 Department of Pediatrics, Sitaram Bhartia Institute of Science and Research, B-16, Qutab Institutional Area, New Delhi 110 016, India; 2 Department of Clinical Epidemiology Sitaram Bhartia Institute of Science and Research, B-16, Qutab Institutional Area, New Delhi 110 016, India; 3 Department of Gynecology and Obstetrics Sitaram Bhartia Institute of Science and Research, B-16, Qutab Institutional Area, New Delhi 110 016, India

## Abstract

**Background:**

Given the postulated advantages of mother generated index (MGI) in incorporating the patients' viewpoint and in the absence of a validated India specific postpartum quality of life assessment tool we proposed to evaluate the utility of an adapted Mother-Generated-Index in assessing postpartum quality of life (PQOL) in India.

**Methods:**

The study was integrated into a community survey conducted in one district of Delhi by two-stage cluster randomized sampling to recruit women who delivered in the last 6 months. PQOL was assessed using MGI. Physical morbidity and Edinburgh- postnatal-depression-scale (EPDS) were also recorded for validation purposes.

**Results:**

All subjects (249 of 282 eligible) participating in the survey were approached for the MGI evaluation which could be administered to 195 subjects due to inadequate comprehension or refusal of consent. A trend towards lower scores in lower socioeconomic stratum was observed (Primary index score-2.9, 3.7 and 4.0 in lower, middle and higher strata; Secondary Index Score-2.6, 3.2 and 3.0 in lower, middle and higher strata). 59.4% mothers had scores suggestive of possible depression (EPDS; n = 172). Primary index score had a good correlation with validator scores like EPDS (p = 0.024) and number of physical problems (p = 0.022) while the secondary index score was only associated with EPDS score (p = 0.020).

**Conclusion:**

The study documents that the MGI, with its inherent advantages, is a potentially useful tool for postpartum quality of life evaluation in India especially in the absence of an alternative pre-validated tool.

## Background

The concept of quality of life (QOL) is complex and subjective. Calman defines it as 'the extent to which hopes and ambitions are matched by experience' [1]. In this context the aim of medical care should be to narrow the gap between a patient's hope and aspirations and what actually happens. Quality of life measurement methods have been seen as an advance in health care outcomes assessment [2]. However the questionnaire based structured approaches have often been criticized for ignoring the patient's viewpoint. Thus the 'Patient Generated Index' was designed as a disease specific quality of life measure which is self completed and patient centered [2]. The tool requires minor modifications to be made disease or culture specific. It has the in-built advantage of allowing the patient to decide the issues important to him/her allowing applicability of the same questionnaire across socio-economic and educational backgrounds. Mother Generated Index (MGI) is one such modified form of the Patient Generated Index designed for assessment of postnatal quality of life [3]. Comprehensive evaluations of postnatal quality of life using the structured questionnaire [4] and MGI [3] based approaches are available from developed countries. In the absence of a validated India specific QOL tool some authors have attempted evaluation of postnatal physical morbidity [5] while others have specifically evaluated postpartum depression [6,7] but none provide a comprehensive, standardized or comparable quality of life evaluation. Given the wide social, cultural and educational diversity in India we hypothesized that MGI could be more useful than a structured questionnaire in QOL evaluation in India and conducted this study to explore utility in QOL evaluation in a community survey in Delhi.

## Methods

Semi structured interviews were conducted on 20 postpartum mothers from the outpatient department of Sitaram Bhartia Institute as a preparatory step prior to the survey. These involved asking the mothers to identify the most important positive and negative areas of their lives and to rate their importance in the post partum period through open ended questions. This was intended to study the comprehensibility of the concept and to formulate a list of areas perceived to be important. All interviews were video recorded and reviewed. It was noted that all interviewed women identified only 2 to 5 areas after much suggestions and prodding by the counsellors. The women were noted to have conceptual difficulty in identifying any areas or aspects of life which were positively affected by the delivery. In light of the findings of the pilot study, existing literature on the subject was reviewed and it was decided to adapt the index to the Indian setting, possibly at the expense of limiting its comparability to other settings. In an attempt to keep the index as simple as possible we decided to follow the scoring and spending point specifications for patient generated index presented by Patel et al [7]. In accordance with the same specifications it was decided to restrict the number of areas identified to six, to keep the scoring points at 10, to allow 12 spending points and to allow the mother and child counselors to administer the index if requested by the subject. To further simplify the concept for administration we decided to allow use of words like problems/areas/issues with the suggested list (as most of the comments were negative or neutral and this was judged to be easier to understand) and to seek 'spending points' in terms of what they wanted to improve the most.

This survey was conducted by two stage cluster randomised sampling to recruit postpartum women who delivered in the last 6 months. In stage 1, two colonies each from 3 predefined strata based on MCD classification of property tax – High (A, B), Middle (C, D) and Low (E, F, G) were selected by simple random sampling [8]. In stage 2, a sequential house-to-house survey was conducted in each selected colony using one of four random directions till all houses were linearly covered or a minimum 50 subjects from the colony meeting the selection criteria and willing to participate in the survey were identified. Details of the study design and sampling have been reported earlier [9]. Selected subjects were then given a date and time for questionnaire administration within 2 weeks of the initial visit. Women who delivered a live viable newborn (after 28 weeks) in last 6 months were included in the survey. Women to whom the survey questionnaire could not be administered (unable to communicate, seriously ill, physical/mental disability), women with major illnesses- cardiac, renal, hepatic, intestinal, neurological disease requiring continuing treatment or has required hospital admission for > 1 week prior to recruitment (within the last one year) and women who had delivered outside Delhi were excluded. A detailed written consent was sought from the subjects. No incentives were given other than free test results of haemoglobin, blood pressure, weight and height measurements (data not presented). The project was approved by the institutional ethics committee.

A standardized pretested questionnaire was administered to the mother which included their age, obstetric history, place and mode of delivery. The complete survey included an assessment of the quality of delivery care services (data not presented), cost of maternity care (data not presented) and a third section on postpartum QOL. The questionnaire was translated into Hindi and back translated into English to allow administration in either language. The QOL section included three related parts. One included the Mother Generated Index (see additional file 1), the second included direct questions on acute and chronic postpartum physical problems (see additional file 2) and the third section included the Edinburgh Postnatal depression Scale (EPDS) (see additional file 3). Details of profession, education and income were also recorded to enable classification of socioeconomic status according to the inflation adjusted (wholesale price index) Kuppuswamy scale (KS) i.e high socioeconomic class (HSEC), middle socioeconomic class (MSEC) and low socioeconomic class (LSEC). A separate consent was sought before administration of the QOL and depression related questions.

The Mother Generated Index is a single sheet three step questionnaire. In step 1 the mother was asked to specify up to five areas of her life that had been influenced/affected by having had a baby. In addition a sixth row is provided to represent all other aspects of life that are not captured in the first five areas. In step 2, she was asked to give herself a score out of 10 for each of these areas. The average of these scores gave the primary index score (PIS) (max = 10; lower PIS ~poorer quality of life). In step 3, she was asked to allocate 12 spending points to improve any one or more of these six areas of life. They were asked to distribute these points in any manner they chose but could not use more or less than 12 points. This was to see the relative importance of potential improvement in the six areas. The overall score also known as the secondary index is calculated by taking weighted sum of each area as specified in example in see additional file 4. The secondary index score (SIS) ranges from 0–10 where 0 reflects that "reality most falls short of patients hopes and expectations" and 10 is the "greatest extent to which reality matches expectations".

Edinburgh postpartum depression scale is depression screening tool with a ten question rating scale with four choices per questions scored from 0 to 3. The maximum possible score is 30 and subjects with a score of ≥ 13 are considered to have likely depression while those with a score of ≥ 10 are considered to have possible depression. As specified EPDS is a screening tool and is not confirmatory. The tenth question on the scale classifies the frequency of suicidal thoughts into 'Yes, Quite often, Sometimes, Hardly ever and Never'.

Data entry and analysis was done using Epi-info2002 and SPSS v 13.0. Complex samples procedure of the SPSS was used to adjust the results for the two stage stratified cluster design of the survey (inter and intra cluster variation). Complex sample linear regression models were used to study the relationship of baseline factors with the primary and secondary index score.

## Results

249 women were recruited (of 282 eligible subjects) from 5279 houses screened in the community. They were interviewed by a mother and child educator between February and April 2007. According to Kuppuswamy scale 77 women were categorized as HSEC, 43 women as MSEC and 129 women as LSEC. All recruited women were approached for the Mother Generated Index evaluation but 43 women from LSEC, 7 women from the MSEC and 4 women from HSEC could not be administered the questionnaire. The reasons included refusal of consent, inability to understand the questionnaire and reluctance to discuss any problems. Thus data on MGI was available for 195 subjects (78.3%). There were no significant differences in demographic characteristics between participants to whom MGI was administered compared with those to whom it could not be administered. Ninety four subjects were administered the questionnaire in Hindi while 101 were administered the questionnaire in English. No significant differences were noted in the mean scores or the areas identified by subjects administered the questionnaire in English or Hindi.

The socio-demographic profile of the subjects is presented in Table 1. The average age of the subjects was 27.0 years and 46.4% mothers were primiparous. Overall 34.6% women had a cesarean section and the rate was 51.8%, 28.1% and 13.8% respectively in the high, middle and low socioeconomic classes.

**Table 1:** Socio demographic profile of the population*

		**According to Socio-economic Class**		
**Characteristics**	**Overall(n = 195)**	**HSEC (n = 73)**	**MSEC (n = 36)**	**LSEC (n = 86)**

**Age (years)^γ^**	27.0(25.8–28.2)	29.2(28.3–30.0)	25.1(23.5–26.6)	24.7(24.2–25.2)

Primi **(%)**	46.4 (36.2–56.8)	51.1 (34.5–67.5)	63.7 (48.3–76.8)	34.1 (30.3–38.2)

**BMI(kg/m^2^)^γ^**	24.8(23.1–26.5)	27.0(25.2–28.8)	24.5(22.7–26.3)	21.9(20.3–23.6)

**Education level (%)^γ^**				

Illiterate/Primary school	15.8 (4.1–45.3)	-	-	42.5 (16.8–73.1)

Middle or High school	31.0 (18.1–47.6)	5.3 (0.3–48.1)	62.4 (30.9–86.0)	54.6 (25.0–81.3)

≥ College education	53.2 (31.7–73.6)	94.7 (51.9–99.7)	37.6 (14.0–69.1)	2.8 (1.7–4.7)

**Gross monthly family income (Rs.)^γ^**				

< 11,750	47.4 (23.9–72.2)	1.8 (0.6–5.7)	72.9 (42.9–90.6)	100 (0.0–100.0)

11,750–23,499	11.5 (3.7–30.7)	16.1 (5.1–40.5)	27.1 (9.4–57.1)	-

23,500–50,000	8.9 (2.5–26.8)	17.8 (6.0–42.4)	-	-

>50,000	32.2 (17.1–52.1)	64.3 (36.6–84.9)	-	-

**Current Employment Status^γ ^(%)**				

Never worked	69.6 (49.9–84.1)	40.6 (30.8–51.3)	96.4 (60.2–99.8)	99.4 (92.0–100.0)

Working full time	8.3 (2.1–27.8)	16.7 (4.2–47.7)	-	-

Working part time	6.7 (2.9–14.7)	12.5 (5.9–24.5)	1.8 (0.1–23.9)	0.6 (0.0–8.0)

Not working at present	15.4 (6.1–33.7)	30.2 (16.5–48.6)	1.8 (0.1–23.9)	-

**Place of delivery**				

Hospital^ψ^	79.0 (57.0–91.4)	80.9 (62.8–91.4)	88.3 (56.0–97.8)	73.2 (33.5–93.6)

Government	36.4 (28.8–44.7)	8.3 (2.0–29.0)	58.3 (53.2–63.2)	66.6 (34.5–88.3)

Private	42.6 (26.4–60.5)	72.6 (58.7–83.1)	30.0 (15.8–49.4)	6.6 (3.2–13.2)

Non- Institutional^π^	12.2 (6.9–20.8)	17.7 (8.0–34.7)	9.9 (2.4–33.5)	5.7 (2.0–15.0)

Home	8.8 (1.3–42.0)	1.4 (0.1–18.4)	1.8 (0.1–23.9)	21.2 (4.6–60.0)

**Mode of Delivery**				

CS	34.6 (19.7–53.3)	51.8 (41.0–62.4)	28.1 (11.6–54.0)	13.8 (4.8–33.5)

Elective CS	58.4 (35.9–78.0)	60.6 (36.7–80.3)	32.0 (2.7–88.9)	65.9 (37.9–86.0)

Emergency CS	41.6 (22.0–64.1)	39.4 (19.7–63.3)	68.0 (11.1–97.3)	34.1 (14.0–62.1)

NVD with Perineum intact	17.9 (5.9–43.5)	1.4 (0.1–18.4)	4.5 (0.3–39.6)	44.7 (23.7–67.8)

NVD with epi	42.8 (36.9–49.0)	43.6 (37.3–50.1)	62.8 (40.9–80.5)	34.9 (20.3–53.0)

NVD with tear	0.9 (0.2–4.4)	0.7 (0.1–7.7)	1.8 (0.1–23.9)	0.9 (0.0–30.0)

Instrumental	3.7 (0.9–14.1)	2.5 (0.4–13.8)	2.7 (0.1–35.2)	5.7 (0.9–28.8)

*Data is presented as cluster adjusted mean (95% CI) or percentage (95% CI)

^γ^These items reflects the status of the women at the time of conducting the survey

^μ^Anemia was defined as Hb =< 11 gm%.

^£^Any OPD or IPD medical reimbursement.

^ψ^Hospital was defined as > 25 beds setup.

^π^Non institutional delivery includes nursing home, private dispensary, government dispensary and individual practitioner home (clinic).

The average primary index score was 3.6 (3.3 to 3.9) while the average secondary index score was 2.9 (2.4 to 3.4) (Table 2). A trend towards lower quality of life scores in lower socioeconomic strata was observed (Primary index score HSEC-4.0, MSEC-3.7, LSEC-2.9 (2.5 to 3.4)), Secondary index score HSEC- 2.5, MSEC-.2.8, LSEC- 2.0). Difficulty in sleeping was the most frequently reported concern in the HSEC and MSEC groups (66.8% (95%CI 49.6 to 80.4) and 64.7 (95%CI 43.7 to 81.3) respectively) while tiredness and physical problems were most commonly reported by the LSEC (72.2% (95%CI 53.8 to 85.3) and 66.9% (95%CI 39.7 to 86.1) respectively) (see additional file 5). In the HSEC, the lowest scores related to emotional disturbances received the worst scores (Mean Score = 2.9), physical problems and tiredness were scored the worst in the MSEC (Mean Score = 2.6 and 2.8 respectively) while weight related concerns, emotional disturbances and financial worries were scored the worst in the LSEC (Mean Score = 0.6, 1.8 and 1.8 respectively). Subjects from the high and middle income groups spent the highest number of spending points on physical problems (Mean spending points = 4.1) and weight related concerns (Mean Spending Points = 3.8) while those from the lower income groups spent most points on financial worries (Mean spending Points = 4.0) (see additional file 5).

**Table 2:** Post partum quality of life (MGI) and EPDS scores by socio economic class*

**OVERALL (n = 195)**		**HSEC (n = 73)**		**MSEC (n = 36)**		**LSEC (n = 86)**	
**Primary Index Score (max = 10; n = 195)**	3.6(3.3 to 3.9)	**Primary Index Score**	4.0(3.4 to 4.6)	**Primary Index Score**	3.7(3.1 to 4.3)	**Primary Index Score**	2.9(2.5 to 3.4)

**Secondary index Score (max = 10; n = 195)**	2.9(2.4 to 3.4)	**Secondary index Score**	3.0(2.4 to 3.7)	**Secondary index Score**	3.2(1.8 to 4.5)	**Secondary index Score**	2.6(1.9 to 3.3)

**EPDS Score (n = 172)**	10.9 (9.7 to 12.0)	**EPDS Score**	9.5 (8.9 to 10.1)	**EPDS Score**	9.3 (5.9 to 12.8)	**EPDS Score**	13.4 (12.8 to 13.9)

*Data is presented as cluster adjusted mean (95% CI) or percentage (95% CI)

Physical problems (24.8%; Mean Score (MS) – 2.1; Mean Spending Points (MSP) – 3.9), work related problems (31.6%; MS – 2.3; MSP – 3.5), baby related concerns (6.2%; MS – 0.0; MSP- 5.0) and financial problems (8.3%; MS- 1.2; MSP- 3.0) were rated the worst (mean score < 3) and reported by significant proportion of mothers (> 5%) of preterm babies (n = 25) compared with physical problems (44.8%; MS-2.4; MSP-3.7) and emotional disturbances (17.7%; MS-2.5; MSP-2.9) in mothers of term babies (n = 170).

The EPDS could be administered to 172 mothers (of 195) of which 59.4% mothers had a score of ≥ 10 (possible depression), 10.9% mothers had suicidal thoughts and 36.6% mothers were suffering from likely depression (defined as score ≥ 13). The incidence of possible depression [HSEC-44.9% (95%CI 30.5 to 60.2), MSEC-51.6% (95%CI 22.1 to 80.1), LSEC-83.7% (95%CI 65.4 to 93.3)], likely depression [HSEC-27.7% (95%CI 22.0 to 34.3), MSEC-22.3% (95%CI 5.8 to 57.0), LSEC-54.4% (95%CI 48.0 to 60.7)] and suicidal thoughts [HSEC-9.0% (95%CI 5.1 to 15.3), MSEC-6.6% (95%CI 0.4 to 53.9), LSEC-15.3% (95%CI 10.4 to 22.0)] was higher in the lower socioeconomic classes.

As reported in Table 3 acute postpartum problems like excessive bleeding were reported more often by the vaginally delivered mothers (4.2% versus 1.0%). The chronic postpartum problems like back pain, tiredness, and inability to do routine duties were reported more often by abdominally delivered mothers. 94% of vaginally delivered mothers and 98.4% mothers in the cesarean group reported no acute postpartum physical complication. The postpartum problems were reported more often by primiparous women (Table 4).

**Table 3:** Distribution of post partum physical problems according to mode of delivery^α^

	Overall (n = 195)	NVD (n = 136)	CS(n = 59)
**Acute Post Partum Physical Complications (%)**			

Inability to pass urine	0.2 (0.0–3.8)	0.4 (0.0–5.5)	0 (0)

Excessive bleeding	3.1 (1.7–5.5)	4.2 (2.0–8.8)	1.0 (0.1–12.1)

Need to remove placenta in OT or stitching in OT	0.6 (0.0–9.3)	0.9 (0.1–13.8)	0(0)

Others	0.6 (0.3–1.3)	0.5 (0.0–9.3)	0.7 (0.0–11.6)

No complication	95.5 (91.6–97.6)	94.0 (86.5–97.5)	98.4 (97.3–99.0)

Subacute/Chronic Post Partum Physical Problems (%)*			

Painful Perineum ^μ^	5.3 (2.9–9.7)	8.1 (3.8–16.4)	-

Fever	2.8 (0.2–27.8)	1.9 (0.1–27.3)	4.5 (0.5–32.5)

Infection from cut/torn perineum ^π ^(n = 100)	2.5 (0.6–9.3)	5.2 (1.4–17.8)	-

Pain at the site of CS ^γ^	3.8 (0.9–14.9)	-	10.9 (2.9–33.7)

Infection at the site CS ^γ^	0.6 (0.3–1.3)	-	1.6 (1.0–2.7)

Urinary incontinence	0.2 (0.0–3.8)	0.4 (0.0–5.5)	0 (0)

Bowel Problems	4.1 (1.0–15.5)	3.9 (0.6–20.2)	4.4 (0.4–33.5)

Sore nipple/breast tenderness	4.0 (0.8–17.5)	5.3 (1.1–21.2)	1.7 (0.1–24.6)

Breast infection	2.0 (0.4–9.7)	2.8 (0.4–15.8)	0.7 (0.0–11.6)

Physical Exhaustion, tiredness	5.0 (2.4–10.1)	2.8 (1.0–7.3)	9.2 (3.1–24.2)

Back pain	8.2 (3.1–20.0)	3.9 (1.4–10.1)	16.3 (4.5–44.5)

Painful Intercourse ^δ ^(n = 92)	2.2 (0.1–30.5)	3.7 (0.2–41.7)	0 (0)

Inability to do routine duties	4.3 (0.8–19.6)	3.6 (0.8–15.2)	5.5 (0.7–31.7)

Relation with partner	1.4 (0.1–11.5)	0 (0)	4.0 (0.5–25.8)

^α^Data is presented as cluster adjusted mean (95% CI) or percentage (95% CI)

*Reported as a "major problem for more than 7 days" in %

^μ^Subjects who had a vaginal delivery

^π^Subjects who had episiotomy or suturing of tear

^γ ^Subjects who had a cesarean section

^δ ^Subjects who had resumed sexual relations since the birth of the baby

**Table 4:** Distribution of post partum physical problems according to parity^α^

	**Primi (n = 92)**	**Multi (n = 103)**
**Acute Post Partum Physical Complications (%)**		

Inability to pass urine	0 (0)	0.4(0.0–6.6)

Excessive bleeding	2.7(0.6–10.9)	3.5(2.6–4.6)

Need to remove placenta in OT or stitching in OT	1.3(0.1–17.1)	0(0)

Others	0.7(0.0–10.3)	0.4(0.0–6.6)

No complication	95.3(82.4–98.9)	95.7(91.5–97.8)

Subacute/Chronic Post Partum Physical Problems (%)*		

Painful Perineum ^μ ^(n = 136)	17.0 (8.9–30.0)	0.9 (0.0–16.4)

Fever	2.6 (0.1–36.4)	2.9 (0.3–23.9)

Infection from cut/torn perineum ^π ^(n = 100)	2.3 (0.2–19.6)	8.6 (1.3–39.8)

Pain at the site of CS ^γ ^(n = 59)	18.2 (5.6–45.4)	3.9 (0.2–42.3)

Infection at the site CS ^γ ^(n = 59)	0 (0)	3.2 (1.6–6.4)

Urinary incontinence	0.5 (0.0–8.3)	0 (0)

Bowel Problems	7.5 (2.3–21.8)	1.1 (0.1–17.6)

Sore nipple/breast tenderness	8.7 (1.7–33.9)	0 (0)

Breast infection	3.9 (0.6–21.5)	0.4 (0.0–6.6)

Physical Exhaustion, tiredness	9.4 (3.7–21.9)	1.3 (0.1–17.6)

Back pain	10.9 (3.9–26.6)	5.9 (1.7–18.0)

Painful Intercourse ^δ ^(n = 92)	4.7 (0.3–47.8)	0 (0)

Inability to do routine duties	9.2 (1.5–39.5)	0 (0)

Relation with partner	0.7 (0.0–10.3)	1.9 (0.1–26.4)

^α^Data is presented as cluster adjusted mean (95% CI) or percentage (95% CI) taking into account South Delhi's demographics

*Reported as a "major problem for more than 7 days" in %

^μ^Subjects who had a vaginal delivery

^π^Subjects who had episiotomy or suturing of tear

^γ ^Subjects who had a cesarean section

^δ ^Subjects who had resumed sexual relations since the birth of the baby

To further explore the utility of MGI we conducted a multivariate regression analysis with Primary and Secondary Index Scores as the dependent variables [3] (Table 5). Possible confounders were identified by review of literature (Mother's age [3], Parity [3], Mode of delivery [3], place of delivery [3], maturity of newborn [4] and employment status of mother [10]) and correlation analysis (No of physical problems, KS Score, Body Mass Index (BMI), EPDS Score). As depicted the number of physical problems and EPDS were significantly associated with the primary index score (p = 0.024 and p = 0.024 respectively) after adjusting for co-variates while the EPDS score was the only significant association of the secondary index score (p = 0.020).

**Table 5:** Regression analysis: Statistical correlates of Mother Generated Index^α^

	**Primary Index score**				**Secondary Index score**			
	
	**Univariate (n = 195)**		**Multivariate^ω ^(n = 172)^§^**		**Univariate (n = 195)**		**Multivariate^ψ ^(n = 172)^§^**	
	
	**β-value**	**p-value**	**β-value**	**p-value**	**β-value**	**p-value**	**β-value**	**p-value**
**Mother's Age**	0.070(-0.072–0.213)	0.241	0.028(-0.096–0.151)	0.567	0.037(-0.091–0.164)	0.471	0.030(-0.078–0.139)	0.481

**No. of Physical problems**	-0.127(-0.202–0.051)	**0.010**	-0.150(-0.267–0.033)	**0.024**	-0.146(-0.342–0.050)	0.107	-0.164(-0.382–0.054)	0.104

**Primi/Multi**	-0.135(-0.408–0.139)	0.243	-0.054(-0.667–0.558)	0.818	-0.334(-0.705–0.038)	0.067	-0.054(-0.817–0.709)	0.854

**BMI**	0.077(0.012–0.142)	**0.030**	0.022(-0.059–0.103)	0.496	0.073(0.002–0.144)	**0.047**	0.064(-0.038–0.165)	0.157

KS Score*	0.056(0.006–0.106)	**0.036**	0.049(-0.026–0.123)	0.143	0.014(-0.049–0.077)	0.574	-0.009(-0.163–0.146)	0.884

**EPDS Score^¶^**	-0.074(-0.121–0.027)	**0.012**	-0.055(-0.098–0.012)	**0.024**	-0.085(-0.150– -0.020)	**0.022**	-0.090(-0.156– -0.023)	**0.020**

**Operative delivery vs. others ^β^**	0.337(-0.611–1.286)	0.379	-0.060(-0.554–0.435)	0.755	-0.130(-1.288–1.029)	0.771	-0.371(-1.448–0.706)	0.393

**Hospital vs. Non Institutional **^π^	0.127(-0.459–0.714)	0.579	0.108(-0.562–0.778)	0.678	-0.175(-1.040–0.690)	0.604	-0.067(-0.595–0.462)	0.744

**Working vs. not working**^μ^	0.261(-0.769–1.291)	0.521	-0.465(-1.179–0.249)	0.145	-0.276(-1.874–1.322)	0.657	-0.331(-2.966–2.303)	0.745

**Premature babies ^δ^**	0.608(-1.500–2.717)	0.468	0.558(-1.007–2.123)	0.378	0.351(-1.816–2.519)	0.676	0.457(-1.078–1.992)	0.455

**Days Since Birth^γ^**	0.001(-0.004–0.006)	0.599	0.000(-0.002–0.001)	0.607	-0.001(-0.009–0.007)	0.741	2.99E(-0.005–0.005)	0.999

^ω^R^2 ^= 0.197 (Model: Primary Index score = Mother's Age + total number of physical problems + parity + Body Mass Index + Kuppuswamy socioeconomic class score + Edinburgh Postnatal depression Scale score + Operative delivery vs. others + Hospital vs. Non Institutional + Working vs. not working + premature babies + Days since birth)

^α^Data is presented as cluster adjusted mean difference in total MGI score(95% CI)

^ψ^R^2 ^= 0.148 (Model: Secondary Index score = Mother's Age + total number of physical problems + parity + Body Mass Index + Kuppuswamy socioeconomic class score + Edinburgh Postnatal depression Scale score + Operative delivery vs. others + Hospital vs. Non Institutional + Working vs. not working + premature babies + Days since birth)

^§ ^For 23 subjects EPDS could not be filled due to refusal of consent. *Kuppuswamy socioeconomic class score (Continuous variable)

^¶ ^Edinburgh Postnatal depression Scale score (Continuous variable)

^β ^Cesarean Section vs. Vaginal delivery (Including NVD, NVD with Episiotomy, Forceps Delivery, Vacuum Delivery)

^π ^Hospital vs. Non Institutional delivery (includes nursing home, government health center and individual practitioner clinic).

^μ^Presently working mothers vs not working

^δ ^Preterm babies (defined as < 37 weeks) vs term

^γ ^Time elapsed since birth at the time of questionnaire administration rounded of to the nearest day.

## Discussion

The study documents that the MGI is a potentially useful tool for quality of life evaluation in post partum women and especially so in the absence of a pre-validated questionnaire. The tool has good criterion validity (correlates well with physical morbidity and validator scores like EPDS), is comprehensive (able to provide information on a wide range of potentially relevant issues) and allows easy administration of general instructions in any language. It has the inherent advantage of determining and rating comments which are deemed important by the subject. However the MGI does not have the intrinsic capability to test for internal reliability unlike structured questionnaires. Also the tool has poor practicality or applicability in the LSEC as the tool could not be completed successfully by a substantial proportion of subjects (33.8%) from the LSEC.

This is the first study evaluating post partum quality of life in India using a standardized, comprehensive and replicable index while documenting the limitations of the method used. However, the study is limited by the poor ability of the subjects from the LSEC to complete the questionnaire. The original mother generated index was modified in the context of the problems observed in the pilot study limiting the comparability of the results to other settings. The primarily negative nature of the comments selected using the pilot study, although necessitated by the conceptual difficulties faced, could be expected to result in lower overall quality of life scores. Also, the study was conducted in one district of a big metropolis limiting the generalizability of the results. Despite the limitations the study provides useful information on the possible utility of the concept in the Indian setting and identifies important issues faced by the mothers in the post partum period.

Several authors from developed countries have evaluated post partum quality of life using structured questionnaires [11,4] and MGI based approaches [3]. The character and expanse of the information provided by the MGI is comparable or better than that reported for structured questionnaires like Maternal Postpartum Quality of Life (MAPP-QOL) [4]. The overall quality of life scores in our study were lower than those reported in other populations using either the MGI or structured questionnaire approach. In a study on 184 women using MAPP-QOL, Hill et al [4] reported that women who have delivered a term infant give the worst scores to the Health and Functioning domain as compared with worst scores for Emotional concerns in mothers who had a preterm child. The mean overall and domain specific scores in this study were much higher than those from our study (20.8/30 compared with 3.6/10 in our study). In our study physical problems, work related concerns, baby related concerns and financial problems were poorly rated and reported by a significant proportion of the mothers (> 5%) of preterm babies compared with physical problems emotional disturbances in mothers of term babies. In a study in the US on 132 women comparing pre and postnatal physical, mental and self rated quality of life scores, significant deterioration was noted in the domains of vitality (p = 0.031), sleep (p = 0.009) and self rated quality of life (p =< 0.001) from the pre to the post natal period [11]. Scores in the domains of general health, vitality, mental health and self-rated quality of life were generally higher than those reported in our study.

Symon AG et al [3] using MGI on 103 women reported that 'tiredness', 'less time to themselves' and 'time with family members' were the most common comments cited by the mothers at 6–8 weeks post partum. In another study by the same author the overall mean primary index score was 4.8/10 in unemployed and 6.3/10 in working mothers [10] compared with 3.5/10 in unemployed mothers and 3.8/10 in working mothers in our study.

As discussed earlier, the overall lower scores in our study could be related to the primarily negative nature of the areas identified in the pilot survey or could reflect a poorer quality of life our subjects. Although it is difficult to be certain on the issue the overall paucity of positive areas identified by the mothers in the pilot study and the subsequently lower overall quality of life ratings during the survey, coupled with the ratings on physical morbidity and EPDS scores do suggest that post partum quality of life in the given population is poorer than that reported in literature from developed countries.

## Conclusion

Postnatal quality of life data from India is scanty and given the absence of a validated structured questionnaire the mother generated index provides a useful and possibly advantageous alternative. The index offers inherent advantages by incorporating the patients' viewpoint, largely avoiding the need for linguistic validation and potentially allowing comparisons across the disparate cultural and lingual heterogeneity of Indian states and across the world. The overall low scores in the current study need confirmation in a wider variety of settings but nonetheless highlight the need for integration of quality of life impact into clinical outcome evaluations in the future especially in developing countries like India where it is often ignored. The possibility of further optimizing the index for the Indian population by reducing the number of items asked deserves exploration. Further work is also necessary to study the correlation of MGI with ethnicity and other validator scores like Post-natal Morbidity Index [PNMI; 12] and Maternal Adjustments and Maternal Attitude [MAMA; 13] scale.

## Abbreviations

MGI: Mother-Generated-Index; PQOL: Postpartum Quality of Life; EPDS: Edinburgh-postnatal-depression-scale; QOL: Quality of Life; KS: KuppuswamyScale; HSEC: High Socioeconomic Class; MSEC: Middle Socioeconomic Class; LSEC: Low Socioeconomic Class; PIS: Primary Index Score; SIS: Secondary Index Score; BMI: Body Mass Index; MAPP-QOL: Maternal Postpartum Quality of Life; PNMI: Post-natal Morbidity Index; MAMA: Maternal Adjustments and Maternal Attitude.

## Competing interests

The authors declare that they have no competing interests.

## Authors' contributions

AB conceived the idea for the survey. JN, RS and SS planned the survey design and supervised the data collection. AS collected the data with the help of a research team. Data was analyzed by JN and AS. RS, SS and AS drafted the manuscript. All authors contributed to the final version of the manuscript. VLB will act as guarantor for the paper.

## Supplementary Material

Additional file 1 **Mother Generated Index.** The mother generated index proforma with the suggestion list and method of scoring. Click here for file

Additional file 2 **Postpartum physical problems.** It includes direct questions on acute and chronic postpartum physical problems. Click here for file

Additional file 3 **Edinburgh Postnatal depression Scale (EPDS).** The EPDS questionnaire and scoring Click here for file

Additional file 4 **Example.** An example demonstrating how to calculate the primary index score and secondary index score Click here for file

Additional file 5 **Table.** MGI scores (Mean (95% CI), spending points (Mean (95% CI) and most common comments of participants (Percentage (95% CI) according to socioeconomic class Click here for file
