# Supplementary material for: An exploratory study to evaluate the utility of an adapted Mother Generated Index (MGI) in assessment of postpartum quality of life in India
Source: Health Qual Life Outcomes. 2008 Dec 2;6:107. doi: 10.1186/1477-7525-6-107 (PMC2651123; doi:10.1186/1477-7525-6-107)
Supplement: Additional file 1 — Mother Generated Index. The mother generated index proforma with the suggestion list and method of scoring. [file 1477-7525-6-107-S1.doc]

**APPENDIX: 1**

**MOTHER GENERATED INDEX-INSTRUCTIONS**

**STAGE 1:** In the boxes below write down five most important areas of your lives affected by the child birth. Here are some areas mentioned that might help you to think how your life has been affected-

Social life, Work, House work, Hobbies, Loss of independence, Makes me moody, Can’t go shopping, Sleep, Tiredness, Finances, Less time with other family members, Physical problems like backache, pain in stitch line, weight gain, Low self esteem, Can’t have pets, Relation with partner, Less time for self

**STAGE 2:** In this part score the areas you mentioned in stage 1.This score should show how badly affected you were over the past month. Please score each area out of 10 using this scale:

10= Exactly as you would like to be

9= Close to how you would like to be

8= Very good but not how you would like to be

7= Good, but not how you would like

6= Between good and fair

5= Fair

4= Between poor and fair

3= Poor but not the worst you could imagine

2= Very poor but not the worst you could imagine

1= Close to the worst you could imagine

0= The worst you could imagine

**STAGE 3:** We want you to imagine that any or all the areas of your life could be improved. You have 12 imaginary points to spend to show which areas you would most like to see improved. Spend more points on areas you would most like to see improve and less on areas that are not so important

STAGE 1-Identified Areas STAGE 2-Scoring each area STAGE 3-Spending points

109876543210

wo

Best

Fair

Worst

All other aspects of your life not mentioned above

109876543210

wo

Best

Fair

Worst

109876543210

wo

Best

Fair

Worst

109876543210

wo

Best

Fair

Worst

109876543210

wo

Best

Fair

Worst

109876543210

wo

Best

Fair

Worst

**MOTHER GENERATED INDEX- SCORING CHART**
