# Supplementary material for: An exploratory study to evaluate the utility of an adapted Mother Generated Index (MGI) in assessment of postpartum quality of life in India
Source: Health Qual Life Outcomes. 2008 Dec 2;6:107. doi: 10.1186/1477-7525-6-107 (PMC2651123; doi:10.1186/1477-7525-6-107)
Supplement: Additional file 2 — Postpartum physical problems. It includes direct questions on acute and chronic postpartum physical problems. [file 1477-7525-6-107-S2.doc]

**APPENDIX-2**

**Q1**. Did you have any immediate postpartum complications?

□ Urinary retention (inability to pass urine)

□ Swelling or collection of blood around the stitches/cut

□ Excessive bleeding

□ Shifted to OT for Manual Removal of Placenta/ traumatic PPH

□ Fecal/Urinary incontinence

□ Others

□ None

**Q2**. Many women have a lot of physical problems in the first few weeks after childbirth. How much of a new problem would you rate the following problems after delivery and time for which the problem lasted? *Remember, if you have given birth more than once please answer the questions as they pertain to the last pregnancy (Q 1205/603)*

| **COMPLAINT** | **RATING** | **DUR (days)** |
| --- | --- | --- |
| Painful perineum |  |  |
| Fever |  |  |
| Infection from cut/ torn Perineum |  |  |
| Pain at site of CS |  |  |
| Infection at site of CS incision |  |  |
| Urinary incontinence |  |  |
| Urinary tract infection |  |  |
| Bowel problems [constipation, incontinence] |  |  |
| Hemorrhoids |  |  |
| Sore nipple/ breast tenderness |  |  |
| Breast infection |  |  |
| Other breast problems[milk supply] |  |  |
| Physical exhaustion, tiredness |  |  |
| Back pain |  |  |
| Thrombosis |  |  |
| Painful intercourse |  |  |
| Inability to get up from bed |  |  |
| Inability to do routine duties |  |  |
| Relationship with partner |  |  |

| **Rating of the Problem** | |
| --- | --- |
| 1 | not a problem |
| 2 | a minor problem |
| 3 | major problem |
| 4 | not sure |

| **Classification of duration** | |
| --- | --- |
| A | < 2 days |
| B | 2-7 days |
| C | 1-4 weeks |
| D | 1-3 months |
| E | > 3 months |
| F | continuing |
