# Supplementary figures and images for: An exploratory study to evaluate the utility of an adapted Mother Generated Index (MGI) in assessment of postpartum quality of life in India
Source: Health Qual Life Outcomes. 2008 Dec 2;6:107. doi: 10.1186/1477-7525-6-107 (PMC2651123; doi:10.1186/1477-7525-6-107)

**APPENDIX 3**

**
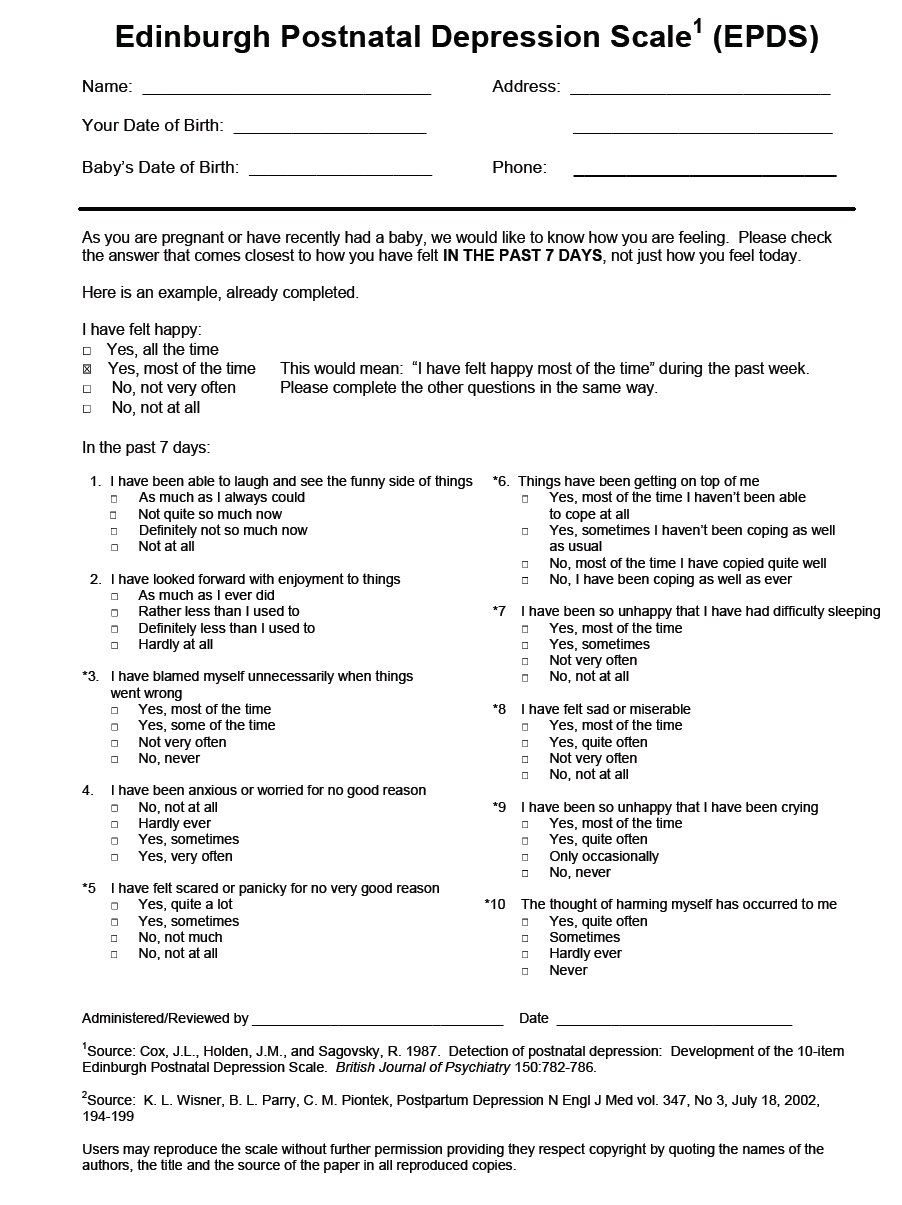
**

**
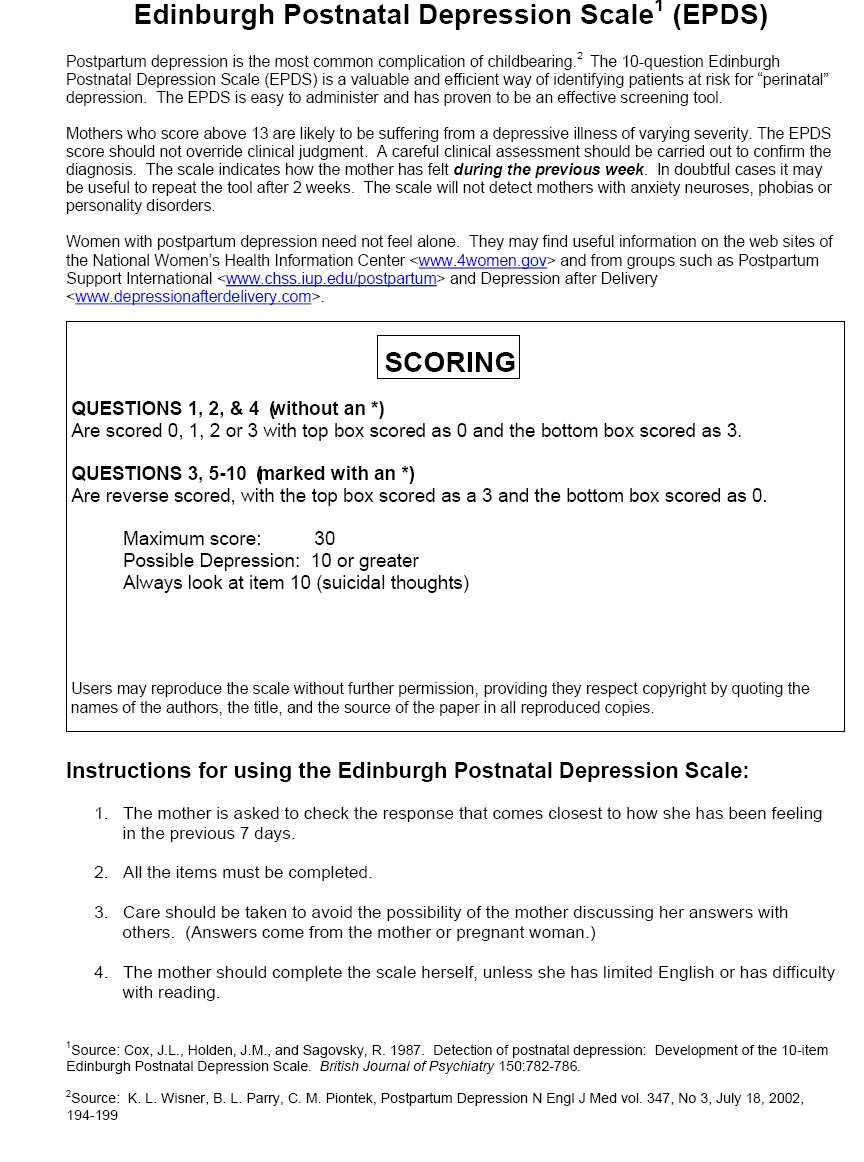
**

Supplement: Additional file 3 — Edinburgh Postnatal depression Scale (EPDS). The EPDS questionnaire and scoring [file 1477-7525-6-107-S3.doc]
