# Supplementary material for: An exploratory study to evaluate the utility of an adapted Mother Generated Index (MGI) in assessment of postpartum quality of life in India
Source: Health Qual Life Outcomes. 2008 Dec 2;6:107. doi: 10.1186/1477-7525-6-107 (PMC2651123; doi:10.1186/1477-7525-6-107)
Supplement: Additional file 4 — Example. An example demonstrating how to calculate the primary index score and secondary index score [file 1477-7525-6-107-S4.doc]

**Appendix 4**

Best

Fair

Worst

109876543210

wo

109876543210

wo

Best

Fair

Worst

109876543210

wo

Best

Fair

Worst

109876543210

wo

Best

Fair

Worst

109876543210

wo

Best

Fair

Worst

STAGE 1-Identified Areas STAGE 2-Scoring each area STAGE 3-Spending points

*Less time for self*

*All other aspects of your life not mentioned above*

1

*Weight gain*

*Difficulty in work*

*Difficulty in sleeping*

*Tiredness*

4

1

1

2

3

- The total Primary Index score for this example can be calculated as:

109876543210

wo

Best

Fair

Worst

[0 + 2 + 3 + 5 + 2 + 5] / 6 = 2.8

- The total Secondary index score for this example can be calculated as:

[0x3+2x4+3x1+5x1+2x2+5x1] / 12

0+8+3+5+4+5/12 = 2.1
