# Supplementary material for: An exploratory study to evaluate the utility of an adapted Mother Generated Index (MGI) in assessment of postpartum quality of life in India
Source: Health Qual Life Outcomes. 2008 Dec 2;6:107. doi: 10.1186/1477-7525-6-107 (PMC2651123; doi:10.1186/1477-7525-6-107)
Supplement: Additional file 5 — Table. MGI scores (Mean (95% CI), spending points (Mean (95% CI) and most common comments of participants (Percentage (95% CI) according to socioeconomic class [file 1477-7525-6-107-S5.doc]

**Additional file 5: MOST COMMON COMMENTS OF PARTICIPANTS BY SOCIOECONOMIC CLASS****

|  | **OVERALL (n=195)** | | | | **HSEC (n=73)** | | | | **MSEC (n=36)** | | | | **LSEC (n=86)** | | | |
| --- | --- | --- | --- | --- | --- | --- | --- | --- | --- | --- | --- | --- | --- | --- | --- | --- |
|  | **Comment*** | **Percentage** | **MS (max=10)^γ^** | **MSP (max=12)^θ^** | **Comment*** | **Percentage** | **MS (max=10)^γ^** | **MSP (max=12) ^θ^** | **Comment*** | **Percentage** | **MS (max=10)^γ^** | **MSP (max=12)^θ^** | **Comment*** | **Percentage** | **MS(max=10)^γ^** | **MSP (max=12) ^θ^** |
| **1.** | Difficulty in sleeping^ρ^ | 64.6(57.0 to 71.6) | 3.7(2.8 to 4.6) | 3.1(2.3 to 3.9) | Difficulty in sleeping^ρ^ | 66.8(49.6 to 80.4) | 3.5(3.0 to 4.1) | 3.1(1.6 to 4.6) | Difficulty in sleeping^ρ^ | 64.7(43.7 to 81.3) | 4.5(4.2 to 4.8) | 4.0(3.5 to 4.5) | Tiredness^µ^ | 72.2(53.8 to 85.3) | 3.0(2.3 to 3.6) | 2.8(2.3 to 3.2) |
| **2.** | Tiredness^µ^ | 53.2(37.8 to 68.0) | 3.5(2.8 to 4.1) | 2.3(1.9 to 2.7) | Restriction of outdoor activities^Ψ^ | 61.8(45.0 to 76.2) | 4.4(3.4 to 5.3) | 1.4(0.4 to 2.5) | Tiredness^µ^ | 48.4(30.2 to 67.1) | 2.8(0.2 to 5.4) | 2.3(1.1 to 3.5) | Physical problems^ν^ | 66.9(39.7 to 86.1) | 1.9(1.2 to 2.6) | 3.5(2.1 to 4.9) |
| **3.** | Difficulty in work^α^ | 51.9(43.1 to 60.7) | 3.9(3.3 to 4.5) | 2.6(2.1 to 3.2) | Less time for self^κ^ | 56.4(44.7 to 67.5) | 4.2(3.3 to 5.1) | 1.7(0.9 to 2.6) | Restriction of outdoor activities^Ψ^ | 48.3(29.7 to 67.3) | 4.6(4.0 to 5.1) | 2.2(1.0 to 3.4) | Difficulty in work^α^ | 64.3(50.1 to 76.4) | 3.9(2.8 to 5.0) | 2.4(2.0 to 2.8) |
| **4.** | Restriction of outdoor activities^Ψ^ | 49.0(33.3 to 65.0) | 4.2(3.6 to 4.9) | 1.7(1.0 to 2.6) | Difficulty in work^α^ | 45.6(31.4 to 60.5) | 4.0(3.3 to 4.7) | 3.0(1.7 to 4.2) | Physical problems^ν^ | 53.7(30.4 to 75.5) | 2.6(1.2 to 4.0) | 4.1(1.3 to 7.0) | Difficulty in sleeping^ρ^ | 61.7(49.4 to 72.6) | 3.6(1.6 to 5.6) | 2.8(2.5 to 3.1) |
| **5.** | Physical problems^ν^ | 42.5(22.3 to 65.7) | 2.4(2.0 to 2.7) | 3.7(2.3 to 5.2) | Tiredness^µ^ | 40.3(25.1 to 57.5) | 4.3(3.2 to 5.5) | 1.8(0.8 to 2.7) | Difficulty in work^α^ | 40.9(24.9 to 58.9) | 3.5(2.1 to 4.9) | 2.5(1.2 to 3.8) | Restriction of outdoor activities^Ψ^ | 32.2(13.6 to 58.7) | 3.7(1.8 to 5.6) | 2.2(1.6 to 2.8) |
| **6.** | Less time for self^κ^ | 40.2(30.0 to 51.2) | 4.1(3.5 to 4.7) | 2.1(1.5 to 2.8) | Less time with husband/family members^λ^ | 34.2(18.3 to 54.6) | 3.9(1.7 to 6.1) | 2.6(1.5 to 3.7) | Less time for self^κ^ | 34.3(22.5 to 48.4) | 4.5(3.4 to 5.7) | 3.4(1.9 to 4.8) | Less time for self^κ^ | 20.4(17.5 to 23.5) | 3.7(3.0 to 4.3) | 2.9(2.7 to 3.1) |
| **7.** | Less time with husband/family members^λ^ | 22.2(13.3 to 34.7) | 3.7(1.9 to 5.5) | 2.8(1.9 to 3.6) | Weight related concerns^ξ^ | 26.8(14.7 to 43.7) | 3.2(2.5 to 4.0) | 3.8(1.3 to 6.3) | Less time with husband/family members^λ^ | 22.7(13.4 to 35.9) | 3.8(0.1 to 7.6) | 3.2(2.3 to 4.2) | Emotional disturbances^π^ | 20.3(8.0 to 42.8) | 1.8(-2.0 to 5.6) | 2.6(2.2 to 3.0) |
| **8.** | Emotional disturbances^π^ | 17.4(10.4 to 27.7) | 2.5(0.8 to 4.2) | 2.7(1.5 to 3.9) | Loss of independence^δ^ | 23.0(7.7 to 51.6) | 3.7(1.1 to 6.3) | 1.8(1.3 to 2.2) | Emotional disturbances^π^ | 11.0(5.6 to 20.4) | 3.8(-0.2 to 7.7) | 2.0(-1.2 to 5.1) | Financial problems^β^ | 13.2(8.1 to 20.6) | 1.8(-0.1 to 3.7) | 4.0(1.4 to 6.5) |
| **9.** | Weight related concerns^ξ^ | 15.7(8.5 to 27.2) | 2.8(1.9 to 3.8) | 3.8(1.6 to 5.9) | Physical problems^ν^ | 21.6(12.5 to 34.6) | 3.2(2.8 to 3.7) | 4.0(1.1 to 7.0) | Financial problems^β^ | 8.0(0.6-55.5) | 3.3(-1.1 to 7.7) | 1.3(-0.8 to 3.5) | Less time with husband/family members^λ^ | 6.0(3.8 to 9.2) | 2.1(0.4 to 3.8) | 3.2(2.4 to 4.0) |
| **10.** | Loss of independence^δ^ | 13.6(4.7 to 33.5) | 3.3(1.5 to 5.1) | 2.0(1.6 to 2.5) | Emotional disturbances^π^ | 16.8(7.2 to 34.6) | 2.9(1.3 to 4.6) | 2.9(0.8 to 4.9) | Other medical problems^σ^ | 5.6(0.6 to 36.9) | 5.0 (1E+29 to -1E+29) | 1.0(1.0 to 1.0) | Weight related concerns^ξ^ | 5.4(3.1 to 9.0) | 0.6(-1.1 to 2.2) | 3.6(2.2 to 5.0) |
|  | All other aspects of life^ω^ | **39.1(20.1 to 62.2)** | **2.1(0.8 to 3.5)** | **0.5(0.1 to 0.9)** | All other aspects of life^ω^ | **73.7(58.4 to 84.9)** | **3.5(2.6 to 4.4)** | **0.9(0.2 to 1.6)** | All other aspects of life^ω^ | **32.0(8.8 to 69.8)** | **1.8(- 0.1 to 3.6)** | **0.3(-0.2 to 0.8)** | All other aspects of life^ω^ | **8.2(5.0 to 13.2)** | **0.4(0.1 to 0.7)** | **0.2(-0.0 to 0.3)** |

**Legend to additional file 5**

**Data is presented as cluster adjusted mean (95% CI) or percentage (95% CI) taking into account South Delhi’s demographics

* This column depicts the ten most common comments reported by the subjects arranged in descending order of frequency.

**^γ^ Mean Score (Scale 0 to 10)**

**^θ^ Mean Spending Points (out of a maximum of 12)**

**^µ^ Includes “weakness” , “dizziness” , “no rest”, “difficulty in walking”, general exhaustion and poor physical fitness**

**^ν^ Includes “bodyache”, “backache”, “stomach ache”, “headache” , “pain in stitches”, “pain in chest”, “eyes pain”, “pain in legs”, “due to pain not able to sit for long”, “Numbness in hands and legs”**

**^α^ Includes “Difficulty in pursuing routine office/ household work which they were able to do earlier”**

**^ρ^ Includes “lack of sleep”, “difficulty in sleeping”, “poor sleep”**

**^Ψ^ Includes “Can’t go out”, “can’t go shopping”, “no time for hobbies”, “lack of time for social life”**

**^π^ Includes ”emotional imbalance”, “anger”, “tension”, “moody“**

**^κ^ Includes ”less time for T.V.”, “can’t study”, “disruption of schedule”, “less time for food”**

**^λ^ Includes “Less time for elder child/children”, “husband or other family members”, “relation with partner”**

**^β I^ncludes “Whether I will be able to give good education to my child” ,”Financial worries”**

**^ξ^ includes concerns about “excessive weight gain” or “weight loss”**

**^δ^ Includes concerns related to lack of independence or freedom or dependence on family members**

**^σ^ includes “Fever”, “Constipation”, “Blood Pressure”, “Urinary Tract Infection”**

**^ω^ Includes all aspects of life which the subject felt contributed to his/her quality of life but could not be accommodated in the five comments the subject was allowed to cite**
